# Temporal variation of chronic child malnutrition in the context of strengthening healthcare services in Burkina Faso: an Oaxaca-Blinder multivariate decomposition analysis

**DOI:** 10.3389/fpubh.2024.1356918

**Published:** 2024-03-26

**Authors:** Pengdewendé Maurice Sawadogo, Jean-François Kobiané, Eric Tchouaket Nguemeleu

**Affiliations:** ^1^Institut Supérieur des Sciences de la Population (Higher Institute of Population Sciences), Joseph Ki-Zerbo University (ISSP/UJKZ), Ouagadougou, Burkina Faso; ^2^Département Sciences Infirmières (Nursing Science Department), University of Quebec in Outaouais, Saint-Jérôme, QC, Canada

**Keywords:** chronic malnutrition, temporal change, Oaxaca-Blinder decomposition, healthcare services, child health, Burkina Faso

## Abstract

Malnutrition seriously affects children’s health, survival, and future productivity. According to the literature, increasing the supply of health services should help reduce the spread of malnutrition. This article analyses the sources of changes in the decline of chronic malnutrition during the 2000s, where there was an increase in the supply of health services in Burkina Faso. We used data from demographic and health surveys conducted in 2003 and 2010 in Burkina Faso. Malnutrition was defined according to the recommendations of the World Health Organization, while using standards of growth which are current and uniform for the two periods of study considered. We analyzed the source of temporal variation of chronic malnutrition through the Oaxaca-Blinder multivariate decomposition of the proportion of children suffering from chronic malnutrition. The analyses showed that the relative extent of chronic malnutrition in children decreased significantly, from 43.4% (CI 95%: 42.3–44.4) in 2003 to 34.7% (CI 95%: 33.6–35.9) in 2010. A quarter of this variation is due to a change in characteristics (composition effect), and the remaining 74.74% is due to a difference in coefficients (performance or behavior effect). Improved access to health services played a crucial role in reducing the scale of chronic malnutrition between 2003 and 2010. Other factors, such as educating mothers and urbanization, also contributed significantly. This study shows that improving access to health services is crucial for reducing chronic malnutrition. So, programs tackling child malnutrition must first and foremost ensure that children have access to health services.

## 1 Introduction

Malnutrition is still a significant threat to the survival and development of children. In developing countries, it accounts for 45% of under-five mortality (1). Chronic malnutrition is the most widespread form, affecting 148.1 million under-five children worldwide in 2023. Its prevalence is highest in Melanesia (46.4%) and Central Africa (37.4%) (2). Furthermore, contrary to the trend observed in other parts of the world, the number of under-five children affected by chronic malnutrition continues to rise sharply in West and Central Africa (2). This continued increase in the number of malnourished children does not support the goal of eliminating chronic malnutrition by 2030 (3).

Temporal variation refers to the change in the extent of chronic malnutrition between two period. According to the scientific literature, factors influencing the temporal variation of child chronic malnutrition are mothers’ access to healthcare (prenatal consultation, assisted childbirth, iron supplementation), child immunization coverage, household purchasing power, mothers’ level of education, access to the media, variations in food prices, fertility and variations in GDP *per capita* (4–9). Although these studies have provided an inventory of factors, the methods used do not allow for estimating the contribution of each factor. Therefore, ranking contribution is necessary to assess more accurately the effects of public policies on improving the state of health of our populations. Furthermore, to our knowledge, no study has analyzed the sources of chronic malnutrition among children in Burkina Faso during the decade 2000.

In Burkina Faso, the chronic malnutrition situation is just as worrying. The national nutrition survey showed that around one child in four suffers from malnutrition in Burkina Faso (10). During the 2000s, Burkina Faso strengthened its health system by increasing health funding, conforming to the Abuja Declaration. The country also adopted a health policy in 2006 and drew up the National Health Development Plan (PNDS) 2001–2010. These policy initiatives have led to a significant increase in the supply and use of health services. For example, the use of general consultation services has tripled, rising from 0.22 contact per inhabitant per year in 2001 to 0.63 in 2010. Over the same period, assisted childbirth coverage rose from 34 to 75% (11, 12). The use of child health services has also increased. For example, the percentage of children aged 12–23 months fully immunized rose from 29% in 1998/1999 to 81% in 2010. The percentage of children receiving treatment for diarrhea also increased from 18 to 48% (13, 14). During the same period, the prevalence of chronic malnutrition remained almost constant, rising from 37% in 1998 to 34.6% in 2010 (13, 14). This study examines the sources of variation in chronic malnutrition in children during the 2000 decade, where we observed a strengthening in health service provision in Burkina Faso.

## 2 Data and methods

We used data from the Demographic and Health Surveys (DHS) conducted in 2003 and 2010. The DHS data are the most complete and disaggregated source on chronic malnutrition in children in Burkina Faso during the study period. Following WHO recommendations, we considered that a child was suffering from chronic malnutrition when the ratio of height to age was less than two (−2) standard deviations from the mean reference value. We obtained these reference values from the parameters (height and age) of children who, given the optimal conditions in which they were born and are growing harmoniously (15). In this article, we used the WHO Growth Standards 2006 version as reference values for estimating the nutritional status of children. This study focused on children aged 6–59 months, who are a group at high risk of malnutrition. The variable to be decomposed is the child’s nutritional status. Variables previously identified as having a significant effect on chronic malnutrition in children were retained as independent variables (16). The complete list of variables used in the analysis and their construction is presented in Table 1.

**Table 1:** Characteristics of the study population in 2003 and 2010.

Characteristics	2003		2010	
	*n*	% (95% CI)	*n*	% (95% CI)
Chronic malnutrition				
Yes	3,574	43.4 (42.3; 44.4)	2,311	34.7 (33.6–35.9)
No	4,662	56.6 (49.9; 52.1)	4,349	65.3 (49.8; 52.2)
Sex				
Male	4,204	51.0 (49.8; 52.2)	3,395	51.0 (49.8; 52.2)
Female	4,032	49.0 (47.9; 50.1)	3,266	49.0 (47.8; 50.2)
Size at birth				
Not short	6,929	84.1 (83.2; 84.8)	5,829	87.5 (86.7; 88.3)
Short	1,307	15.9 (15.1; 16.7)	831	12.5 (11.7; 13.3)
Gemellity				
Single	8,055	97.8 (97.4; 98.1)	6,461	97.0 (96.6; 97.4)
Twin	181	2.2 (01.8; 02.6)	199	3.0 (02.6; 03.4)
Minimal meal diversification				
No	2,233	90.3 (89.1; 91.4)	1746	85.1 (83.5; 86.6)
Yes	240	9.7 (08.5; 10.9)	304	14.9 (13.4; 16.4)
Minimal frequency of meals				
No	1710	69.1 (67.2; 70.9)	1,285	62.7 (60.5; 64.7)
Yes	763	30.9 (29.1; 32.7)	765	37.3 (35.2; 39.4)
Pentavalent 3 vaccinated				
No	3,740	51.4 (50.2; 52.5)	1,075	14.8 (13.9; 15.7)
Yes	3,533	48.6 (47.4; 49.7)	4,870	85.2 (84.3; 86.1)
Mother educational level				
None	7,243	87.9 (87.2; 88.6)	5,579	83.8 (82.9; 84.7)
Primary	670	8.1 (07.5; 08.7)	747	11.2 (10.4; 11.9)
Secondary level and above	323	3.9 (03.4; 04.3)	334	5.0 (04.5; 05.6)
Mother’s involvement in decision-making				
None	4,987	60.5 (59.4; 61.5)	3,784	56.8 (55.6; 58.0)
Intermediate	2,921	35.5 (34.5; 36.5)	2,477	37.2 (36.0; 38.4)
Strong	328	4.0 (03.6; 04.4)	399	6.0 (05.4; 06.7)
Mother’s tolerance of violence				
None	1992	24.2 (23.2; 25.1)	3,450	51.8 (50.6; 53.0)
Intermediate	3,494	42.4 (41.3; 43.4)	2,244	33.7 (32.6; 34.8)
Strong	2,750	33.4 (32.4; 34.4)	966	14.5 (13.7; 15.4)
Mother nutritional status				
BMI <18.5	1,402	17.0 (16.2; 17.8)	870	13.1 (12.3; 13.9)
18.5 < =BMI < 25	6,278	76.2 (75.2; 77.1)	5,220	78.4 (77.4; 79.4)
BMI > =25	556	6.8 (06.2; 07.4)	570	8.5 (07.8; 09.2)
Number of children under five in the household				
1–2 children	4,962	60.3 (59.2; 61.3)	4,312	64.7 (63.5; 65.8)
3–4 children	2,297	27.9 (26.9; 28.9)	1886	28.3 (27.2; 29.4)
5 children and more	976	11.8 (11.1; 12.5)	463	6.9 (06.3; 07.5)
Toilet facilities in the household				
No	6,028	73.2 (72.2; 74.2)	4,593	69.0 (67.9; 70.1)
Yes	2,208	26.8 (25.8; 27.8)	2067	31.0 (29.9; 32.1)
Access to improved source of drinking water				
No	7,088	86.1 (85.3; 86.8)	5,545	83.2 (82.2; 84.1)
Yes	1,148	13.9 (13.2; 14.7)	1,115	16.8 (15.9; 17.7)
Household wealth status				
Poor	3,139	38.1 (37.1; 39.1)	2,513	37.7 (36.5; 38.9)
Middle income	2,852	34.6 (33.6; 35.6)	2,498	37.5 (36.3; 38.7)
Riche	2,245	27.3 (26.3; 28.3)	1,649	24.8 (23.7; 25.7)
Place of residence				
Urban	1,099	13.4 (12.6; 14.1)	1,125	16.9 (16.0; 17.8)
Rural	7,137	86.6 (85.8; 87.3)	5,535	83.1 (82.2; 84.0)
Average distance to a basic health facility (km)		8.4		7.4

Burkina Faso.

CI, Confidence interval.

Constructed using data from EDSBF 2003 and EDSBF 2010.

The source of temporal variation in chronic malnutrition was analyzed using a multivariate decomposition of the proportion of children suffering from chronic malnutrition. In its simplest form, the decomposition allows us to distinguish between sources of change attributable to a change in the structure of the population (composition effect) and those linked to changes in behavior of the population (17). The decomposition method of Oaxaca and Blinder as adopted by Powers et al., (18) was used in this study to identify the contributions of various factors to the change in the extent of chronic malnutrition between 2003 and 2010 in Burkina Faso. Considering Y as the N x l vector of the dependent variable, X the N x K matrix of the independent variables and β a K x l vector of the regression coefficients. The difference in the proportion of the extent of chronic malnutrition between two periods A and B (2003 and 2010 respectively) is expressed as follows:


$$\bar{Y_{A}}-\bar{Y_{B}}=\bar{F\left(X_{A}\beta_{A}\right)}-\;\bar{F(X_{B}\beta_{B}})\text{.}$$

After adjusting this equation, we obtain:


$$\bar{Y_{A}}-\bar{Y_{B}}=\bar{F(X_{A}\beta_{A}}-\;\bar{F\left(X_{B}\beta_{A}\right)}+\bar{F(X_{B}\beta_{A}})-\;\bar{F\left(X_{B}\beta_{B}\right)}$$

$\bar{F\left(X_{A}\beta_{A}\right)}$-$\bar{\;F(X_{B}\beta_{A}}$) represents the proportion attributable to differences in characteristics and is denoted E. $\bar{F(X_{B}\beta_{A}}$-$\bar{\;F\left(X_{B}\beta_{B}\right)}$ represents the part due to differences in the coefficients and is denoted C. Details of the decomposition equation can be found in the article by Powers et al. (18).

### 2.1 Ethical considerations

This research is based exclusively on secondary data. Ethical requirements were taken into account when selecting data sources. We obtained authorization to access the demographic and health survey databases via an online request to the Macro International website (www.dhsprogramm.com).

## 3 Results

### 3.1 Characteristics of the study population in 2003 and 2010

Table 1 shows the characteristics of the study population in 2003 and 2010. It shows that 43.4% of children under five suffered from chronic malnutrition in 2003, compared with 34.7% in 2010. The proportion of children vaccinated almost doubled, rising from 49 to 85% over the same period. Similarly, the proportion of children from households with improved toilets rose by four percentage points, and those from the ones with access to an improved source of drinking water increased by three percentage points. In addition, mothers’ level of education and autonomy improved significantly between 2003 and 2010.

### 3.2 Sources of variation in the extent of chronic malnutrition

The results of Oaxaca and Blinder’s multivariate decomposition present the effects of change attributable to variation in individual characteristics (E) and the effects of change attributable to the difference in coefficients (C). Overall, the change in the extent of chronic malnutrition between 2003 and 2010 is estimated at 0.102 or 10.2% (Table 2).

**Table 2:** Results of the Oaxaca-Blinder multivariate decomposition analysis of the change in the proportion of children aged 6–59 months between 2003 and 2010.

Characteristics	Differences in characteristics (E)		Differences in coefficients (C)	
	Coefficients	%^a^	Coefficients	%^a^
Age (month)	0.0010***	0.98	0.0602***	58.78
Sex	−0.0002***	−0.20	0.0109	
Size at birth	0.0021***	2.05	0.0012	
Gemellity	−0.0016***	−1.56	−0.0014	
Minimal meal diversification	0.0005		0.0044**	4.30
Pentavalent 3 vaccinated	−0.0049		−0.0192	
Primary education level and above	0.0020***	1.95	−0.0031	
Secondary education level and above	0.0022***	2.15	0.0027	
Mother’s intermediate involvement in decision-making	0.0002		−0.0046	
Mother’s strong involvement in decision-making	0.0001		0.0022	
Mother’s intermediate tolerance of violence	−0.0027**	−2.63	−0.0153**	−14.94
Mother’s strong tolerance of violence	−0.0016		−0.0077**	−7.52
Mother’s normal nutritional status (18.5 < =BMI < 25)	0.0002		0.0329	
overweight or obese mothers (BMI > =25)	0.0019***	1.85	0.0031	
Number of children under five in the household				
3–4 children	0.0003	0.29	−0.0063	
5 children and more	0.0017		−0.0022	
Household with toilet facilities	0.0000		−0.0224	
Household middle income status	−0.0004		−0.0039	
Household rich income status	−0.0018***	−1.76	−0.0064	
Urban residence	0.0057***	5.56	+0.1199**	117.09
Average distance to a basic health facility	0.0191***	18.65	+0.0399	
Constant			−0.1337	
Total	0.0258***	25.26	0.0765***	74.74

**p < 5%; ***p < 1%.

Constructed using data from EDSBF 2003 and EDSBF 2010.

#### 3.2.1 Contribution of the change in characteristics

Overall, a change in the characteristics of the study population contributed to reducing the extent of chronic malnutrition by 2.6 percentage points, representing 25.26% of the total variation between 2003 and 2020. In the same vein, the contributions linked to the increase in the urban population (5.6%) and the ones linked to the increase in the proportion of educated mothers (1.95% for primary education and 2.15% for secondary education) are also significant. On the other hand, negative contributions linked to the variation in the proportion of children of mothers with an average tolerance of violence (−2.6%), the proportion of wealthy households (−1.8%) and the proportion of twins (−1.95%) were also observed (Table 2).

#### 3.2.2 Contribution of the change in coefficients

The change in coefficients (C) contributed to a reduction in the relative extent of chronic malnutrition of 7.7 percentage points, corresponding to 74.74% of the total change. The analysis shows that changes in the coefficients for urbanization (+117%) and child age (+59%) contributed significantly to a reduction in the scale of chronic malnutrition between 2003 and 2010. However, changes in the coefficients associated with moderate (−15%) and high (−7%) tolerance of violence contributed significantly, but in the opposite direction to the trend observed between 2003 and 2010.

## 4 Discussion

**Figure 1:**
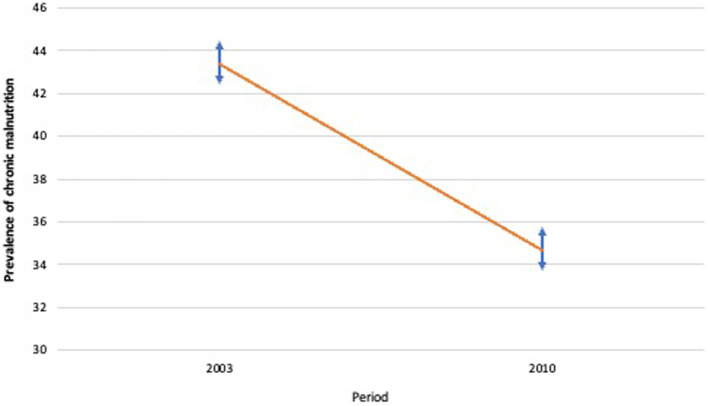
Trend in variation in child chronic malnutrition 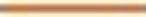 confidence interval 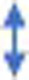.

The relative magnitude of chronic malnutrition in children decreased significantly over the period 2003–2010, from 43.4% [42.3; 44.4] in 2003 to 34.7% [33.6–35.9] (Figure 1). The Oaxaca-Blinder multivariate decomposition showed that 25.26% of the variation in the extent of chronic malnutrition is due to a change in characteristics (composition effect), and the remaining 74.74% is due to a change in coefficients (performance or behavior effect).

### 4.1 Compositional effects

With regard to the variation attributable to the compositional effect, it appeared that the improvement in the population’s geographical accessibility to health services made a significant contribution, accounting for 18.6% of the total variation in the extent of chronic malnutrition between 2003 and 2010. At the national level, 282 new health facilities were built, contributing to reduce the average distance to a basic health facility from 8.68 km in 2003 to 7.34 km in 2010 (12, 19). With this closer proximity to health centers, women and children have greater access to preventive care (vaccination, micronutrient supplementation, monitoring of growth and weight) and curative care in the event of illness, which contributes to reducing the extent of chronic malnutrition in children. Previous studies conducted in sub-Saharan Africa have also shown similar effects, in particular a significant effect of vaccination and access to healthcare (6, 20, 21). Given its strong contribution to reducing the extent of chronic malnutrition, improving geographical accessibility to healthcare services could play an important role in achieving the objective of eliminating chronic malnutrition in children in Burkina Faso by 2030, given that the average radius of action is continuing to be reduced at a rate almost similar to that observed over the 2000s decade (12, 22).

The increase in the proportion of the population living in cities generated the second largest compositional effect on the change in chronic malnutrition between 2003 and 2010. i.e. 5.6% of the total change. A similar significant effect of urbanization was highlighted during the 1990s in Burkina Faso (23). However, the contribution was relatively modest, as the proportion of urban households increased by only 3.5 percentage points between the two periods (Table 1). In addition, the growth of the urban population over the period was followed by an expansion of informal settlements whose inhabitants do not have access to basic social services (water, sanitation, health centers) (24, 25). As a result, a large proportion of the inhabitants of large cities such as Ouagadougou do not have access to basic urban facilities, which should be beneficial for the healthy growth of children.

The increase in the proportion of mothers’ educational level provided the third most significant contribution to the decline in the scale of chronic malnutrition between 2003 and 2010. Taking into account both the change in the proportion of mothers with primary education and the one in the proportion of those with secondary education and above, the overall contribution represents 4%. This result is similar to that of previous studies conducted in Ethiopia, Ghana, Nigeria and Liberia, which found that improving the educational level of mothers significantly contributed to reducing the prevalence of chronic malnutrition (6, 20, 26, 27). Considering the scientific literature, we would expect a greater contribution from mothers’ education, since it has helped to reduce child mortality by more than 50% in developing countries (28). In our case, its contribution to reducing the prevalence of chronic malnutrition has been relatively small as the increase in the proportion of mothers educated to the secondary level has been very small (+1 percentage point). However, only education up to a secondary level has the greatest effect on chronic malnutrition in children (29, 30). This result shows that pursuing policies that encourage girls to attend school in Burkina Faso could significantly contribute to reducing the prevalence of chronic malnutrition.

### 4.2 Behavioral effects

Most of the variation in the extent of chronic malnutrition is due to changes in behavior. Those changes in behavior are mainly associated with urbanization and the age of the child. The change in behavior associated with urbanization has made a major contribution to the reduction in chronic malnutrition in children, i.e., by 117%. This strong contribution can be explained by the reduction in inequalities in health-promoting behavior between urban and rural areas. With the significant increase in health coverage in rural areas (31), rural women’s knowledge of health and nutrition has been raised. In addition, programs and projects have used communication sessions to boost rural women’s knowledge. Consequently, the gap between rural and urban areas has narrowed so that place of residence, a determining factor in chronic child malnutrition in 2003, was no longer a factor in 2010 (16).

Behavior change associated with children’s age contributed 59% in reducing the extent of chronic malnutrition in children between 2003 and 2010. This effect could reflect a reduction in vulnerability to disease with age. Empirical studies and theoretical frameworks show that the frequency of illness is a major factor in chronic malnutrition in children (32–35). This vulnerability to disease is closely linked to the child’s age. During the first six months of life, the child benefits from the protection of maternal antibodies and is therefore relatively less susceptible to disease. Between 12 and 23 months of age, the child is more vulnerable to disease since the maternal antibodies have almost disappeared and the child’s immune system is still weak. By the age of 24 months, the child’s immune system becomes more mature and he or she is still relatively protected against early childhood illnesses. With the increase in health coverage, children of all ages are benefiting from vaccination and vitamin A supplementation, which strengthens their immune system and means that they are less frequently ill. As a result, children’s age-related vulnerability to diseases, including chronic malnutrition, is reduced, hence the behavioral effect highlighted in this study. In summary, the residence or the age-related behavioral effect reflect the effects of projects and programs in improving access to health services, which have substantially improved the health and nutritional behavior of rural people and reduced the frequency of illness among children.

Although this study produced original results, we should acknowledge a shortcoming in assessing the effects of accessibility to health services. The indicator used -the theoretical average radius of action of health facilities- does not allow for a detailed assessment of geographical accessibility to health services. The use of geographical information tools to calculate the distance (as the crow flies) between each household and the nearest health center should make it possible to estimate these effects more accurately. Analyses of this kind, which were not carried out as part of this study due to the lack of data, could provide further avenues for this research. Finally, the proportion of temporal variation in chronic malnutrition attributable to a change in structural characteristics (education, wealth status) is relatively small. Given that these factors change slowly (inertia of demographic phenomena), it is understandable that the observation period of 7 years (between 2003 and 2010) is insufficient to observe significant demographic changes capable of having a considerable effect on the scale of chronic malnutrition. Analysis over a longer period is needed to better assess the effects of changes in population structure on the development of chronic malnutrition in children.

## 5 Conclusion

This study has shown that an improved provision of healthcare services constituted a significant contribution in reducing the prevalence of chronic malnutrition in children between 2003 and 2010. As a result, the population has had closer access to health services, and rural children, in particular, have had greater access to care, thus protecting them from illnesses that are detrimental to their healthy growth. Similarly, health and nutrition programs have helped to increase the adoption by mothers and households of behaviors conducive to healthy child growth. These results show that projects and programs to combat chronic malnutrition must first and foremost work to increase access to health services.

## Data availability statement

Publicly available datasets were analyzed in this study. This data can be found at: https://dhsprogram.com/data/available-datasets.cfm.

## Author contributions

PS: Writing – original draft, Methodology, Formal analysis, Conceptualization. J-FK: Writing – review & editing, Conceptualization. ET: Writing – review & editing, Conceptualization.
