# Supplementary material for: Temporal variation of chronic child malnutrition in the context of strengthening healthcare services in Burkina Faso: an Oaxaca-Blinder multivariate decomposition analysis
Source: Front Public Health. 2024 Mar 26;12:1356918. doi: 10.3389/fpubh.2024.1356918 (PMC11002249; doi:10.3389/fpubh.2024.1356918)
Supplement: Supplementary file 1 [file Table_1.DOCX]

Annex A1: A description of the construction of the variables used in this study

| - **Variable *** | **Construction** | **Response rate 2003** | **Response rate 2010** |
| --- | --- | --- | --- |
| - Child chronic malnutrition | - A child is considered chronically malnourished (yes modality (1)) when his height-for-age index is less than -2 standard deviations (-2SD) ^[[1]](#footnote-1)^. Modalities are: Yes (1) ; No (0) | - 100% | - 100% |
| - Sex of child | - Modalities are : Male; Female | - 100% | - 100% |
| - Size at birth | - This is the answer to the question "When (NAME) was born, was he/she very big, bigger than average, average, smaller than average or very small?" All children whose mothers judged " shorter than average " or " very short " were classified as " short at birth " and the others as " not short ". | - 100% | - 100% |
| - Gemellity | - Modalities are : Single; Twin | - 100% | - 100% |
| Minimal meal diversification | - Children whose meals are considered diversified are those who have consumed at least 4 of the 7 food groups that are i) breast milk; ii) cereals, roots and tubers; iii) legumes and nuts; iv) dairy products; v) meat products and eggs; vi) vitamin A-rich fruits and vegetables; and vii) other fruits and vegetables in the last 24 hours prior to the surveyors' visit (Global Nutrition Monitoring Framework, 2018)(Cadre mondial de suivi de la nutrition, 2018). Modalities are: Yes (1) ; No (0) | - 100% | - 100% |
| Minimal frequency of meals | The minimum frequency of meals for breastfed children is twice a day for those aged 6-8 months and three times a day for those aged 9-23 months. For non-breastfed children, on the other hand, the minimum required frequency is four times a day for children aged 6-23 months. (Croft et al., 2018). Children whose diet does not meet these conditions are classified as not having the minimum frequency. Modalities are: Yes (1) ; No (0) | - 100% | - 100% |
| - Pentavalent 3 vaccinated | - Children up to date with Pentavalent 3 are those who have received all three doses of the vaccine against Diphtheria, Tetanus, Pertussis, Hepatitis B, Hemophilus Influenza B and Poliomyelitis. Modalities are: Yes (1) ; No (0) | - 100% | - 100% |
| - Age (months) |  | - 100% | - 100% |
| - Mother educational level | - Modalities considered are : None ; primary ; secondary level and above | - 100% | - 100% |
| - Mother's involvement in decision-making | - Mothers' involvement in decision-making was assessed using an index that took into account i) the use of money earned by the woman; ii) the pursuit of health care for herself; iv) major household acquisitions; following a methodology adapted from Bhagowalia, Menon, Quisumbing, and Soundararajan (2012) . Modalities considered are : None (0), Intermediate (1-2), Strong (3) | - 100% | - 100% |
| Mother's tolerance of violence | It is a score constructed from a five-item index through which women find it justified (or not) for a husband to beat his wife in the following situations: i) she goes out without telling her husband, ii) neglects the children, iii) argues with him, iv) refuses to have sex with him or v) burns food. Modalities considered are: None (0), Intermediate (1-3), Strong | 100% | 100% |
| - Mother nutritional status (BMI) | - Built in accordance with WHO recommendations (2000a). Modalities considered are: BMI < 18,5 ; 18.5 <=BMI<25; BMI >=25 | - 99,58 | - 99,7 |
| Number of children under five in the household | Modalities considered are : 1-2 ; 3-4 ; 5 and above | 99,7 | 99,6 |
| Presence of improved toilet facilities in the household | - Flush toilets (piped sewer, septic tank, latrine or unspecified), ventilated aerated pit toilets, pit latrines with slab and compost toilets are considered improved (Croft et al., 2018). Modalities are: Yes (1) ; No (0) | - 100% | - 100% |
| Access to improved source of drinking water | Improved water sources include piped water (from the yard or neighborhood), public taps, boreholes, protected spring water and bottled water (Croft et al., 2018). Modalities are: Yes (1) ; No (0) | 100% | 100% |
| Household wealth status | The three classes are delimited by the two terciles of the index of household economic well-being available in the EDS databases.  Modalities are: Poor ; middle income ; rich | 100% | 100% |

Annex A1: A description of the construction of the variables used in this study (continued)

| Place of residence | Modalities are : Urban, rural | 100% | 100% |
| --- | --- | --- | --- |
| - Average distance to a basic health | - This is the average theoretical radius of action (RMAT) of the health facilities in the province's district(s). It is obtained from the Ministry of Health's statistical yearbooks. It expresses the average distance a household in the province has to travel to reach a first-level health facility. When the province has several districts, we consider the average RMAT level of the districts.. | - 100% | - 100% |

1. children whose z-score is less than -6 standard deviations or greater than +6 standard deviations have been excluded in accordance with the DHS program methodological guide. (Croft et al., 2018) [↑](#footnote-ref-1)
